# Effects of Polybutylene Succinate Content on the Rheological Properties of Polylactic Acid/Polybutylene Succinate Blends and the Characteristics of Their Fibers

**DOI:** 10.3390/ma17030662

**Published:** 2024-01-29

**Authors:** Ik Sung Choi, Young Kwang Kim, Seong Hui Hong, Hye-Jin Seo, Sung-Ho Hwang, Jongwon Kim, Sang Kyoo Lim

**Affiliations:** 1 International Cooperation Team, Korea Textile Development Institute, Daegu 41842, Republic of Korea; ischoi@textile.or.kr; 2 Department of Fiber System Engineering, Yeungnam University, Gyeongsan 38541, Republic of Korea; 3 Department of Energy Technology, Daegu Gyeongbuk Institute of Science and Technology (DGIST), Daegu 42988, Republic of Korea; kimyk1211@dgist.ac.kr (Y.K.K.); kjyhsh@dgist.ac.kr (S.H.H.); seohaejin511@dgist.ac.kr (H.-J.S.); hsungho@dgist.ac.kr (S.-H.H.); 4 Department of Interdisciplinary Engineering, Daegu Gyeongbuk Institute of Science and Technology (DGIST), Daegu 42988, Republic of Korea

**Keywords:** rheology, mechanical property, polybutylene succinate, polylactic acid

## Abstract

Polylactic acid (PLA) and polybutylene succinate (PBS) are gaining prominence as environmentally friendly alternatives to petroleum-based polymers due to their inherent biodegradability. For their textile applications, this research is focused on exploring the effects of PBS content on the rheological properties of PLA/PBS blends and the characteristics of PLA/PBS blend fibers. PLA/PBS blends and fibers with varying PBS contents (0 to 10 wt.%) were prepared using melt-blending and spinning methods. Uniform morphologies of the PLA/PBS blends indicated that PBS was compatible with PLA, except at 10% PBS content, where phase separation occurred. The introduction of PBS reduced the complex viscosity of the blends, influencing fiber properties. Notably, PLA/PBS fibers with 7% PBS exhibited improved crystallinity, orientation factor, and elasticity (~16.58%), with a similar tensile strength to PLA fiber (~3.58 MPa). The results suggest that an optimal amount of PBS enhances alignment along the drawing direction and improves the molecular motion in PLA/PBS blend fiber. This study highlights the potential of strategically blending PBS to improve PLA fiber characteristics, promising advancement in textile applications.

## 1. Introduction

Growing concerns regarding plastic waste disposal have propelled a recent upsurge in interest in bioplastics. This interest stems from the urgent need to seek alternatives to landfills, with biodegradable polymers emerging as potential solutions [1]. Additionally, the quest to replace fossil fuel-based resources has directed attention toward bio-based thermoplastics such as polylactic acid, polybutylene succinate, polyhydroxyalkanoates, cellulose derivatives, and palm oil-based thermosets, which present viable alternatives to non-degradable petroleum-based polymers [2,3]. PLA, one type of thermoplastic aliphatic polyester produced from renewable resources like plant-based materials rather than fossil fuel, boasts numerous advantages, including accessible raw materials, easy processability, commendable biocompatibility, and biodegradability [4]. However, it grapples with inherent shortcomings, notably brittleness, poor toughness, low melt strength, slow crystallization rates, and limited heat resistance, constraining its further applications [3,4].

There are two kinds of strategies aimed at overcoming the inherent limitation of PLA: surface modification and bulk modification of PLA [5]. Surface-modification strategies for polylactic acid (PLA) have been extensively explored, involving the introduction of reactive groups such as –COOH, –OH, and –NH_2_, and non-reactive groups like –C–O–C– through permanent and non-permanent techniques. Despite successfully achieving controlled wettability, degradation rate, and functionality, minimizing adverse effects on PLA’s bulk properties is critical. Prolonged UV-induced photo grafting, for example, can lead to monomer migration into the film bulk. To mitigate this, it is essential to significantly reduce grafting times, potentially using a high-power UV lamp, while taking precautions such as employing a Pyrex container to prevent PLA degradation under intense UV irradiation. A faster photo grafting process holds promise for making this surface-modification technique commercially viable within practical timeframes.

In contrast, bulk-modification strategies primarily aim to enhance the toughness of PLA, often at the expense of decreased tensile strength, modulus, and degradability. The challenge is to achieve durable toughening without compromising these crucial properties. There is also a concern about toughened PLA gradually losing toughness over time. Balancing these factors presents a significant challenge in developing modified PLA materials with enhanced toughness while preserving other essential characteristics.

Bulk modification can be achieved by incorporating plasticizers, nucleating agents, inorganic fillers, or other toughening materials such as PCL, PBS [6], PBAT, or PE [7]. Additionally, studies have applied thermoplastic polyurethane [8,9] to enhance its properties. Among them, PBS is a commercially available aliphatic polymer known for its excellent flexibility and toughness, often used to counteract the brittleness of PLA [5]. Recently, PBS has been commonly produced through the polycondensation of bio-based 1,4-butanediol with succinic acid sourced from renewable resources. Some studies have indicated that adding PBS can enhance the impact strength and crystallinity of PLA/PBS blends. This improvement is attributed to PBS acting as a nucleating agent [10,11]. PBS also exhibits susceptibility to degradation by the microorganism Amycolatopsis sp. In a liquid medium, *M. rosea* has demonstrated the ability to degrade 50% (*w*/*v*) of PBS film within 8 days [12]. In the context of PLA/PBS blends, prior research highlights the significant influence of PBS on degradation characteristics. Soil burial tests revealed that blends with higher PBS content exhibited accelerated disintegration over 60 days, resulting in approximately 6% and 12% weight losses in PLA/PBS (80/20) and PLA/PBS (20/80) blends, respectively. Gel permeation chromatography revealed a reduction in the average molecular weight over time, which was attributed to the chemical hydrolysis of both PLA and PBS. Enzymatic degradation studies using proteinase K on benzoyl peroxide-modified PLA/PBS blend films further emphasized the impact, showcasing notable changes in surface morphology and a 67% degradation rate within 96 h [13].

With this in mind, numerous attempts have been made to modify the properties of PLA using renewable counterparts. However, a substantial knowledge gap persists in fabricating PLA/PBS fibers and optimizing key parameters to achieve the desired properties in the textile application of PLA/PBS fibers. Therefore, investigating the rheological properties of PLA/PBS blends and the characteristics of PLA/PBS blend fibers holds significant value.

The combination of PLA and PBS in blending presents an intriguing avenue for enhancing the mechanical and environmental characteristics of the resultant fibers. This blending process holds promise in improving the flexibility of PLA/PBS composite fibers, adjusting the melting point, and reducing rigidity (Table 1). Furthermore, the potential improvement of biodegradability at lower temperatures through this blending method introduces an environmental aspect to the material’s characteristics. Combining these effects creates a versatile material with enhanced flexibility, customized thermal properties, and ecological sustainability [13,14].

This study delves into the exploration of PLA and PBS blending, shedding light on the potential advancements in material properties and the subsequent suitability for diverse applications. We produced PLA/PBS blend fibers by blending PLA and PBS using a melt-spinning technique, adjusting the weight ratios of PLA and PBS. To investigate the effect of PBS on the characteristics of the PLA/PBS blend fibers, a thorough analysis was carried out, including the examination of morphology, mechanical properties, and thermal characteristics, with a focus on evaluating the degree of crystallinity. This observation highlights the potential of strategically blending PBS to enhance PLA fiber mechanical properties and crystallization rate for textile applications. This study provides valuable insights into fabricating and improving PLA/PBS blend fibers, offering pathways for optimizing these blends to meet diverse textile application requirements and advancing sustainable polymer materials.

## 2. Materials and Methods

### 2.1. Materials

Poly(lactic acid) (PLA) (Luminy^®^ LX-175) with a density of 1.24 g cm^−3^ and a melt flow index (MFI) of 6 g/10 min (210 °C, 2.16 kg) was purchased from Total Energies Corbion (Gorinchem, The Netherlands). PBS (BioPBS) with a density of 1.26 g cm^−3^ was purchased from Mitsubishi Chemical Group (Tokyo, Japan). Bis (2,6-diisopropyl phenyl) carbodiimide with a molecular weight of 362.61 g mol^−1^ as compatibilizer (ZIKA-AH362) was provided by ZIKO Co., Ltd. (Seoul, Republic of Korea).

### 2.2. Preparation of PLA/PBS Blends

**Figure 1:**
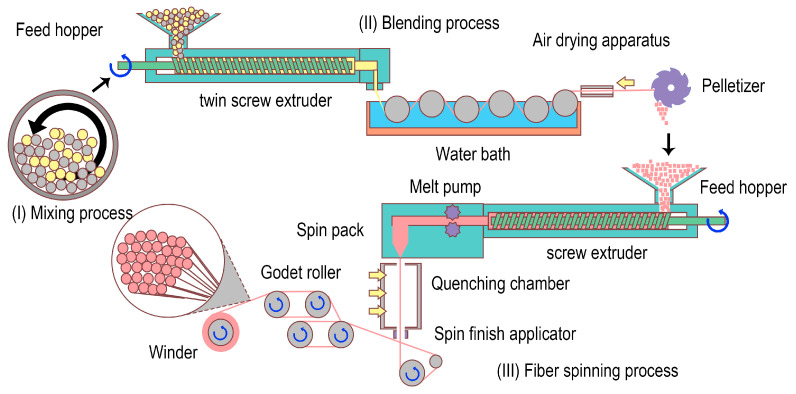
Schematic illustration of the fabrication processes of PLA/PBS blend fibers.

PLA and PBS pellets were dried in a vacuum oven at 80 °C for 4 h before processing to remove moisture. Subsequently, PLA and PBS pellets were mixed with different weight ratios of 100:0, 97:3, 95:5, 93:7, and 90:10 (Figure 1). The compatibilizer was then added in an amount of 0.5 wt.% relative to the weight of the PLA pellets, using the ball mill method at a speed of 50 rpm for 30 min [11]. Following this, blending was carried out employing a co-rotating twin-screw extruder (Haake Polylab OS/RheoDrive 7, Thermo Scientific, Waltham, MA, USA) equipped with a mono-hole spinneret with a circular cross-sectional shape (diameter = 3 mm). The six heating zones within the extruder were adjusted to temperatures of 175, 180, 180, 185, 185, and 180 °C, respectively, and the extrusion speed was 45 rpm. After cooling down in a water bath and drying with air, the extruded filaments were cut into fine pellets using a pelletizer (Thermo Fisher Scientific, Staffordshire, UK). Lastly, the specimens underwent a drying process in an oven at 80 °C for 4 h to eliminate any remaining moisture. The samples were designated as PLA, PLA/PBS 3%, PLA/PBS 5%, PLA/PBS 7%, PLA/PBS 10%, and PBS according to the added contents of PBS relative to PLA, respectively. When the PBS content exceeded 10 wt.% relative to PLA, preparing the PLA/PBS blend fiber became unfeasible due to the decreased mechanical stability of the undrawn yarn. This resulted in failures during the drawing and winding processes of the fibers. Therefore, we chose a PBS proportion ranging between 3 and 10 wt.% in the blend.

### 2.3. Preparation of PLA/PBS Blend Fibers

To evaluate the influence of the PBS content on the characteristics of the PLA/PBS blend fibers, we prepared PLA fibers incorporating PBS using the melt-spinning technique with an extruder equipped with a 36-hole spinneret (FET-MS-LAB 3, Fiber Extrusion Technology Ltd., Leeds, UK). Extrusion temperatures were established by taking into account the rheological characteristics and melt viscosity of the blends. The optimal temperatures of the eight heating zones within the extruder were adjusted to temperatures of 210, 215, 220, 230, 235, 235, 235, and 235 °C. The extrusion speed was 8 rpm. The fibers underwent drawing using five godet rollers and a winder, achieving speeds of 1400, 1700, 2250, 2550, and 2560 mpm, resulting in a draw ratio of 1.8. This process was conducted at the temperatures of 70, 70, 75, 85, 25, and 25 °C, respectively. Subsequently, the fiber was wound at the speed of 2600 mpm. Then, the PLA/PBS blend fibers with 75 denier/36 filaments were acquired. The PBS fiber was prepared with a procedure similar to that used for PLA/PBS blend fibers except for the extruder temperature of 190 °C, godet roller speed of 2900 mpm, and winding speed of 3000 mpm.

### 2.4. Characterization of PLA/PBS Blends and PLA/PBS Blend Fibers

The melt flow index (MFI) of PLA, PBS, and the PLA/PBS blends was assessed utilizing a melt flow indexer (QM280A, Qmesys Co., Ltd., Anyang-si, Gyeonggi-do, Republic of Korea) at the temperature range of melt spinning, specifically 190, 210, and 230 °C, under a load of 2.16 kg. Each sample was measured five times, and the average value was obtained to represent the data.

Rheological experiments were conducted utilizing an MCR 702 rotational rheometer (Anton Paar, Graz, Austria), which was equipped with parallel disks measuring 25 mm in diameter and spaced 1 mm apart. Small amplitude oscillatory shear (SAOS) melt rheology tests were conducted to assess the storage and loss modulus as well as the complex viscosity at a temperature of 230 °C. Frequency sweep experiments were executed at a strain amplitude of 1% and spanning low to high frequencies (0.1~100 rad s^−1^) within the linear viscoelastic range.

High-resolution images of the PLA/PBS blends and PLA/PBS fibers were acquired through the use of a scanning electron microscope (SEM, SU8020, Hitachi, Tokyo, Japan).

Fourier transform infrared (FT-IR) spectra were acquired directly on the PLA/PBS blends using a Bruker Tensor 27 spectrometer connected to an attenuated total reflection accessory. Each spectrum was collected with 32 scans in the range of 4000~600 cm^−1^ at a resolution of 4 cm^−1^.

Dynamic mechanical analysis (DMA) was conducted employing a DMA 850 (TA instruments, New Castle, DE, USA). All specimens were prepared with a size of 12 × 10 × 0.5 mm and subjected to measurements at a heating rate of 3 °C min^−1^ within a temperature range of −50 to 100 °C. The tests were carried out in the air at a frequency of 1 Hz.

Differential scanning calorimetry (DSC) was conducted utilizing a Discovery DSC (TA instruments, New Castle, DE, USA) to analyze the thermal characteristics of both PLA and the PLA/PBS blends. The tests were carried out with a consistent heating and cooling rate of 5 °C min^−1^ under a nitrogen flow of 50 mL min^−1^. In the initial DSC scan, the temperature was increased from 50 to 170 °C, and maintained at 170 °C for 3 min. Then, it was cooled to 50 °C and held at that temperature for 3 min. This heating–cooling cycle was repeated. The first heating scan was utilized for the DSC analysis of PLA/PBS blend fibers, whereas the second heating scan was employed for the DSC analysis of the PLA/PBS blends.

The degree of crystallinity was calculated using the following equations to identify the effect of PBS content in the PLA/PBS blends on the crystallinity of the PLA/PBS blends.

(1) $$\chi_{c}^{PLA}\left(\%\right)=\left[\frac{\Delta H_{m}^{PLA\;}-\Delta H_{cc}^{PLA}}{\Delta H_{m}^{PLA,0}\times {\;W}^{PLA}}\right]\times 100$$

(2) $$\chi_{c}^{PBS}\left(\%\right)=\left[\frac{\Delta H_{m}^{PBS}}{\Delta H_{m}^{PBS,0}\times W^{PBS}}\right]\times 100$$

where *W*^PLA^ and *W*^PBS^ represent the weight fractions of PLA and PBS in the blend. $\Delta H_{cc}^{PLA}\;$ refers to PLA’s cold crystallization enthalpy during the exothermic process. $\Delta H_{m}^{PLA}$ and $\Delta H_{m}^{PBS}$ denote the heat of fusion for PLA and PBS, while $\Delta H_{m}^{PLA,0}$ and $\Delta H_{m}^{PBS,0}$ are the heat of fusion for 100% PLA and PBS crystals, given as $\Delta H_{m}^{PLA,0}$ = 93.0 J g^−1^ and $\Delta H_{m}^{PBS,0}$ = 210.0 J g^−1^, respectively [15].

Two-dimensional wide-angle X-ray diffraction (2D-WAXD) measurements were conducted using a high-resolution two-dimensional X-ray diffractometer (Bruker D8 Discover, Cu Kα-radiation) to characterize the crystallinity of the PLA fibers. The X-ray beam, with a wavelength of 0.15406 nm, was focused to an area of 4 × 4 μm^2^, and the sample-to-detector distance was maintained at 100 mm during the 2D-WAXD measurements. For collecting 2D-WAXD images, an X-ray 2D detector (Vantec 500, a resolution of 512 × 512 pixels, Bruker, Billerica, MA, USA) was utilized. The crystallinity of PLA/PBS fiber, determined from 1D-WAXD profiles, was calculated using the following equation.

(3) $$\chi_{c}^{PLA}\left(\%\right)=\left[\frac{\sum A_{hkl}^{PLA}}{{\sum A}_{all}}\right]\times 100$$

where $\sum A_{hkl}^{PLA}$ represents the sum of the integrated area of the peak of the PLA crystalline phase, while ${\sum A}_{all}$ signifies the sum of the integrated area across all peaks.

The mechanical characteristics of PLA fibers were examined through a universal testing machine (5565A, Instron, Norwood, MA, USA). Following the preparation of five specimens for each sample, tensile strength was measured at a crosshead speed of 300 mm∙min^−1^ and a gauge length of 50 mm.

## 3. Results and Discussion

### 3.1. Characterization of PLA/PBS Blends

#### 3.1.1. Rheological Properties of PLA/PBS Blends

The rheological properties of a molten polymer at a specific temperature give an insight into optimizing the fabrication conditions (i.e., heating temperature and extrusion speed) for binary blending fibers. In this study, the MFI was measured to assess the melt flow tendency of the PLA/PBS blends in the possible temperature ranges of the melt-spinning process from 190 °C to 230 °C. Table 2 displays the MFI values for PLA, PBS, and PLA/PBS blends. Despite the increasing content of PBS, which exhibits a relatively high MFI of 22.62 g/10 min at 190 °C, the MFI values of all PLA/PBS blends maintain a consistent MFI value just within a 3 wt.% range compared to that of the PLA (3.36 g/10 min) at 190 °C. PLA/PBS 10% exhibits the highest MFI of 13.61 g/10 min at 230 °C. However, the value indicated that the melt-spinning process of PLA/PBS 10% blend fiber can be performed uniformly even at 230 °C. Therefore, the highest melt-spinning temperature of PLA and PLA/PBS blending fiber was set at 230 ± 5 °C, while for PBS fiber, it was established at 190 ± 5 °C.

**Figure 2:**
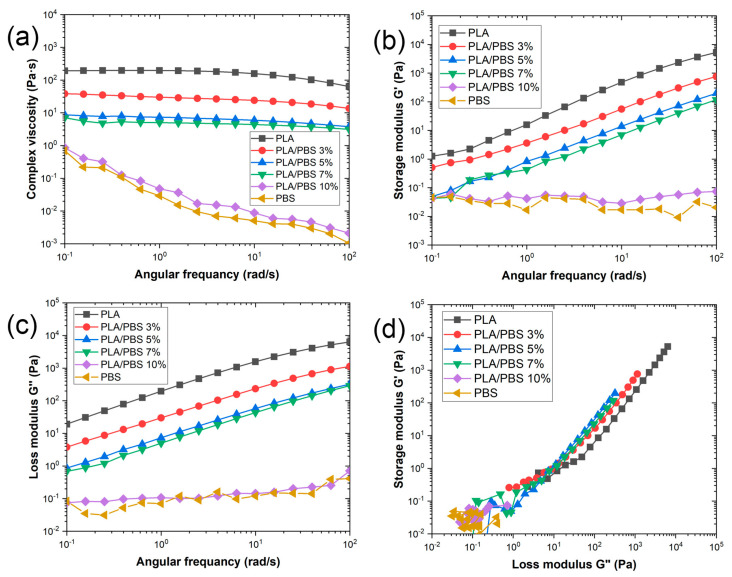
Dynamic rheological properties of PLA, PBS, and the PLA/PBS blends. The graphs of (**a**) complex viscosity, (**b**) storage modulus, (**c**) loss modulus, and (**d**) Han plot: the storage modulus versus loss modulus for the PLA/PBS blends at 230 °C.

In addition to MFI, dynamic shear rheological measurements were conducted on PLA, PBS, and PLA/PBS to verify the viscoelastic properties of the PLA/PBS blends. An amplitude sweep test was executed at a temperature ensuring comparable shear viscosities among blends. Additionally, the assessment encompassed measuring the complex viscosity within the 0.1 to 100 rad s^−1^ range, examining the behavior across wider deformation ranges. The viscoelastic characteristics were evaluated within the linear viscoelastic range under a 1% strain. Figure 2 displays the complex viscosity (η*), storage modulus (G′), loss modulus (G″), and Han plot of PLA, PBS, and the PLA/PBS blends with varying PBS content (3 wt.%, 5 wt.%, 7 wt.%, and 10 wt.%).

The complex viscosity decreased throughout the whole angular frequency range as the PBS content in the PLA/PBS blends increased. Specifically, at 1 rad s^−1^, pertinent to the melt spinning process, the complex viscosity decrease was observed in the following order: PLA > PLA/PBS 3% > PLA/PBS 5% > PLA/PBS 7% > PLA/PBS 10% > PBS, exhibiting values of 196, 54, 9.56, 8.21, 0.0951, and 0.0841 Pa·s, respectively. In Figure 2a, PLA, PLA/PBS 3%, PLA/PBS 5%, and PLA/PBS 7% displayed Newtonian fluid behavior within the low-frequency range of 0.1 to 10 rad s^−1^. Moreover, the complex viscosity showed a gradual decline in the high-frequency range, a characteristic feature of representative rheological behavior in linear polymers. Conversely, the PLA/PBS 10% blend and PBS exhibited shear thinning behavior.

Figure 2b,c show the storage modulus (G′) and loss modulus (G″) as functions of angular frequency for PLA, PBS, and the PLA/PBS blends. In comparison to PLA, the G′ and G″ of the PLA/PBS 3%, PLA/PBS 5%, and PLA/PBS 7% blends showed decreased values across all frequencies. It is evident that all PLA/PBS blends display a typical viscoelastic behavior (G″ > G′) across the entire frequency range [12]. The storage modulus of the PLA/PBS blends is lower than that of PLA, mainly attributed to the inclusion of PBS, which improves molecular motion. The viscosity can be defined with the power law model as the following equation.

(4) $$\eta^{*}=K\cdot {\dot{\gamma }}^{n-1}$$

where *K* represents a constant prefactor, *n* is the power law exponent, and $\dot{\gamma }$ denotes the shear rate. The parameters derived from the power law model are presented in Table 3. These values were simulated using the fitting program in origin software (OriginPro 2020, OriginLab Corporation, Northampton, MA, USA). Adding PBS in PLA reduced the melt viscosity, which was consistent with the earlier analysis of complex viscosity. Additionally, the *n* value of the PLA/PBS blends decreased in comparison to that of PLA, signifying a notable tendency towards shear thinning.

In Figure 2d, a Han plot is depicted, illustrating the correlation between the storage modulus and the loss modulus. The change in the slope of the Han plot indicates changes in the viscoelasticity of the blend. A decrease in the slope (log G′/log G″) signifies an increase in elasticity [16]. As the PBS content rises, the slope of the Han plot for the PLA/PBS blends gradually diminishes, indicating an elevation in elasticity.

#### 3.1.2. Morphology of PLA/PBS Blends

**Figure 3:**
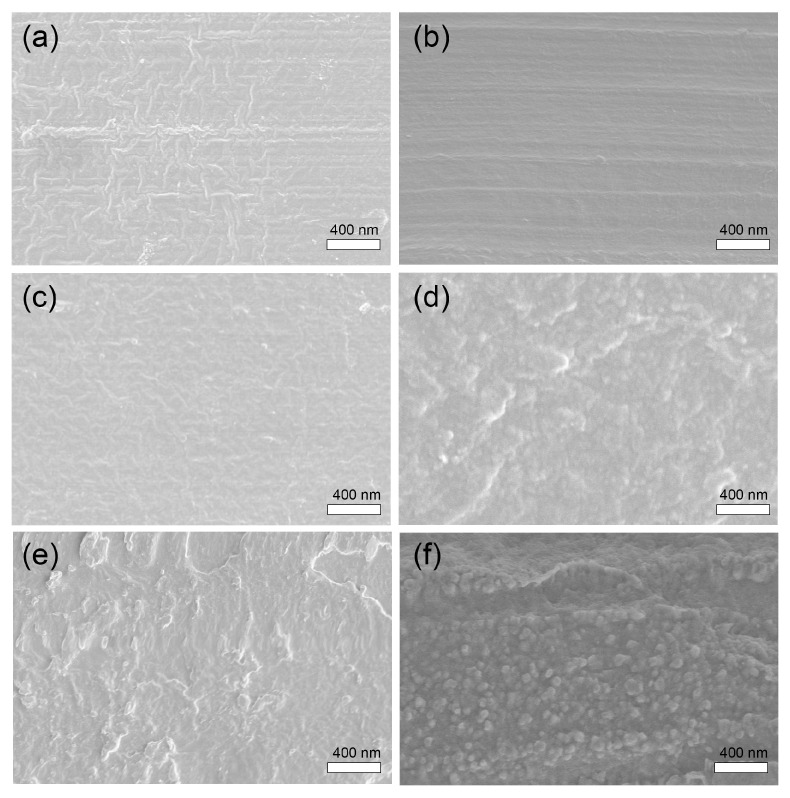
SEM images of the fractured surface of the PLA/PBS blends: (**a**) PLA, (**b**) PBS, (**c**) PLA/PBS 3%, (**d**) PLA/PBS 5%, (**e**) PLA/PBS 7%, and (**f**) PLA/PBS 10%.

The phase morphology of extruded blends significantly influences the formation and mechanical properties of PLA/PBS blend fibers. Typically, the binary blends, being thermodynamically incompatible, exhibit a distinct two-phase structure, resulting in insufficient interactions with the matrix, which is a primary obstacle in fabricating binary blend fibers. Therefore, optimizing both the processing conditions and the phase morphology of binary blends is crucial. The SEM micrographs in Figure 3 display fractured surfaces of PLA/PBS blends with varying PBS contents. Both PLA and PBS demonstrate smooth surfaces. In the case where the PBS content increases to 7 wt.%, rougher surfaces form due to the compatibilizer effectively improving miscibility between PBS and PLA, resulting in a co-continuous phase. However, at a 10 wt.% PBS content in PLA, a notable phase separation between PBS and PLA becomes evident. This separation manifests as a ’sea-island’ structure, where discrete droplets of the PBS phase disperse within the PLA matrix without establishing obvious interfacial bonding. This lack of bonding primarily stems from thermodynamic incompatibility [5] and the high interfacial tension, approximately 3.7 mN m^−1^, between PLA and PBS [17,18]. Due to the phase separation and less adhesion between PBS and PLA in the PLA/PBS 10% blend, we anticipate the mechanical properties of the PLA/PBS 10% blend fiber, especially tensile strength, to be inferior to those of other PLA/PBS blend fibers [19].

#### 3.1.3. FT-IR Analysis of PLA/PBS Blends

Through the analysis of functional group changes in the PLA/PBS blends using FT-IR, the objective was to uncover the reasons for the molecular structural differences resulting from the addition of PBS to PLA and to estimate how PLA interacts with PBS.

**Figure 4:**
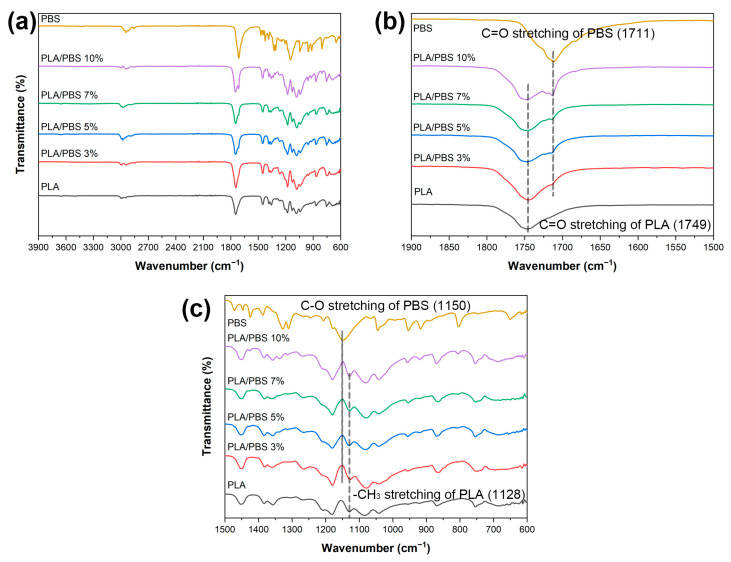
(**a**–**c**) Fourier transform infrared spectra of PLA, PBS, and the PLA/PBS blends.

Figure 4 illustrates the FT-IR spectra featuring PLA, PBS, and PLA/PBS blends. Characteristic peaks within the PBS spectrum, representing diverse molecular bonds and functional groups at distinct wave numbers, are evident. These peaks include –C–OH bending related to the carboxylic acid group at 917 cm^−1^, –O–C–C– stretching vibrations between 1044 and 1046 cm^−1^, and the stretching of the –C–O–C– group in ester linkages ranging from 1144 to 1264 cm^−1^ [20]. Moreover, observations reveal symmetric deformation vibrations of the –CH_2_– group at 1330 cm^−1^. Subsequently, C=O stretching vibrations attributed to the ester group in PBS emerge between 1710 and 1713 cm^−1^, followed by an asymmetric deformational vibration of the –CH_2_-group within the PBS main chains at 2945 cm^−1^ [21]. Finally, a peak at 3430 cm^−1^ is observed, attributed to the –OH stretching vibration of PBS [22].

Moving to the PLA spectrum, notable changes become apparent. Peaks are initially observed at 3750 cm^−1^ (representing –OH stretching vibration), 2946 cm^−1^ (representing a symmetric stretching vibration of CH_3_ in C–H), 1749 cm^−1^ (indicating C=O stretching in the ester group), and a discernible peak at approximately 1085 cm^−1^ signifies C–O–C stretching within the PLA structure [23]. These vibrations are noteworthy when comparing the FT-IR spectra of the PLA/PBS blends. With a PBS addition of up to 3 wt.%, similar vibrations to those of PLA are observed. However, in the case of the PLA/PBS 5%, PLA/PBS 7%, and PLA/PBS 10% blends, characteristic peaks of both PLA and PBS appear, featuring two carbonyl peaks at 1749 and 1711 cm^−1^, respectively. As the PBS content increases up to 7 wt.%, slight variations occur in interactions within the folded PLA chains and the -OH groups in the PBS chain, around 1128 cm^−1^ and 1150 cm^−1^, respectively [24]. These changes indicate strengthened bond energy within the molecular structures, reflecting robust intermolecular interactions, including forming hydrogen bonds between the oxygen functional groups in PLA and PBS. In this case, the compatibilizer may contribute to reinforcing intermolecular interaction. However, the persistence of the unchanged peak at the highest content of PBS in PLA/PBS 10% suggests a reduced interaction between PLA and PBS. This decline could be attributed to the increased presence of PBS, which may lead to a decrease in the overall compatibility between PLA and PBS despite the presence of a compatibilizer. This result aligns with our observations from SEM, the phase separation.

#### 3.1.4. DMA Analysis of PLA/PBS Blends

Dynamic mechanical analysis was utilized to investigate the impact of polymer compatibility on viscoelastic properties. Our study aimed to elucidate the correlation between polymer interactions and the viscoelastic behavior of the PLA/PBS blends. The analysis focused on the changes in storage modulus, loss modulus, and tan δ to temperature.

The storage modulus, defining material stiffness and the capacity for elastic energy storage during deformation, was examined alongside the loss modulus, which characterizes energy dissipation as heat. Additionally, the ratio of dissipated energy to stored energy, represented by tan δ, provided insights into the glass transition temperature of the PLA/PBS blends [25].

**Figure 5:**
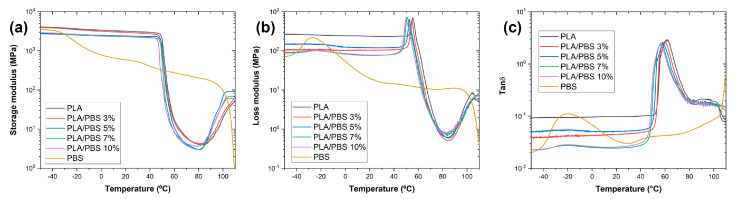
Dynamic mechanical curves of the PLA/PBS blends. (**a**) storage modulus; (**b**) loss modulus; and (**c**) tan δ.

In Figure 5a, within the glass transition region, the storage modulus of PLA measured approximately 4061 MPa. However, when blending with PBS, the storage modulus of the PLA/PBS blends decreased to 2917 MPa (for PLA/PBS 10%). A comparable trend was evident in the loss modulus. The loss modulus of the PLA/PBS blends decreased to 87 MPa from 190 MPa observed for PLA. These behaviors were related to the increased molecular mobility attributed to the lower T_g_ of PBS. Noteworthy variations in storage modulus were observed, specifically in regions similar to the glass transition and cold crystallization. A decline was observed during the glass transition, while an increase was noticeable in the cold crystallization of the PLA/PBS blends. The storage modulus curve for PLA decreased between 43 °C and 84 °C, followed by a subsequent rise. However, for the PLA/PBS blends, the storage modulus decreased within the temperature range of 40 °C to 80 °C, followed by a subsequent rise. The earlier initiation of the rise in storage modulus at lower temperatures was attributed to the increased PBS content in the blend. It indicated that the addition of PBS promotes the cold crystallization of PLA. Furthermore, the PLA/PBS blends exhibited dual glass transition peaks. As the content of PBS increased, the tan δ peak shifted from 61.6 °C (typical for PLA) to 55.94 °C, potentially linked to PBS influencing the dominant free volume [26]. The flexibility of the PBS chain likely facilitated the movement of PLA molecular chains. Conversely, the tan δ peak of PBS shifted from −19.6 °C to −18.7 °C.

The observed shift in T_g_ values because of the blending of PLA and PBS signified compatibility within the blends, suggesting a substantial influence of PBS on the dominant free volume [26]. These findings provided valuable insights into the compatibility of PLA/PBS blends.

#### 3.1.5. Thermal Properties and Crystallinity of PLA/PBS Blends

**Figure 6:**
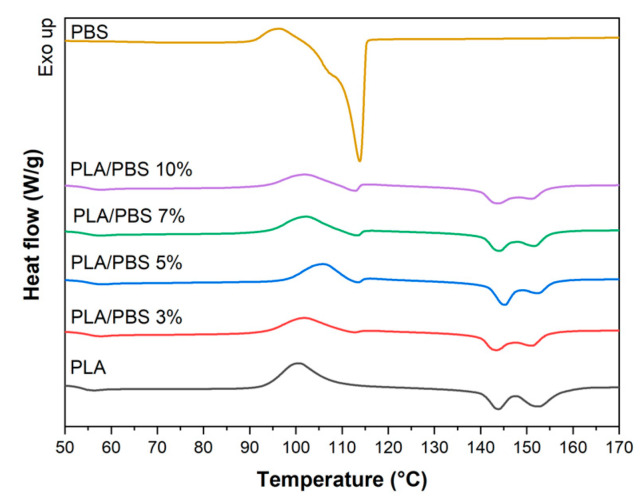
DSC curves of the PLA/PBS blends.

DSC analysis is reliable for evaluating semi-crystalline polymers’ thermal properties and crystallization. Figure 6 displays DSC diagrams for PLA, PBS, and their blends. Regarding the endothermal process, double peaks appear in PLA at approximately 144 and 152 °C. In comparison, PBS exhibits a single melting peak at 113 °C. The other blends show two separate melting peaks, which are observed in their endothermal process, occurring at 112~115 °C and 143~155 °C, respectively. The glass transition temperature of the PLA/PBS blends closely corresponds to that of PLA (Table 4). However, as the PBS content increases to 5 wt.%, an elevation is observed in the cold crystallization temperature, melting temperature, and the degree of crystallinity. The additional incorporation of PBS results in a reduction in these values. Particularly noteworthy is the significant improvement in the degree of crystallinity in PLA/PBS 5% (13.27%) compared to PLA alone (3.84%), marking a 3.46-fold increase. This enhancement is attributed to both the carbodiimide-based compatibilizer and the uniform PBS phase in the blends. The former likely enhances the link between PLA and PBS, while the latter serves as the nucleus for crystallization.

### 3.2. Characterization of PLA/PBS Blend Fibers

#### 3.2.1. Cross-Sectional Morphology of PLA/PBS Blend Fibers

**Figure 7:**
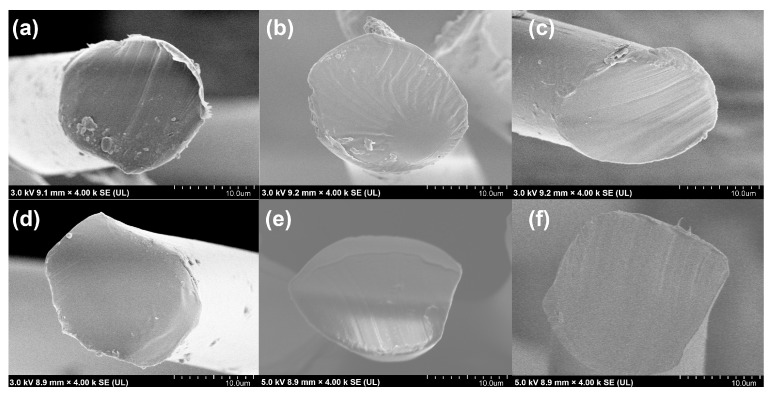
Cross-sectional morphology of the PLA/PBS blend fibers. (**a**) PLA, (**b**) PLA/PBS 3% fiber, (**c**) PLA/PBS 5% fiber, (**d**) PLA/PBS 7% fiber, (**e**) PLA/PBS 10% fiber, and (**f**) PBS fiber.

Figure 7 shows the cross-sectional morphology of the PLA/PBS blend fibers. The diameters of the PLA and PBS fibers are 16.93 μm and 21.41 μm, respectively. The PLA/PBS blend fibers maintain a uniform diameter of approximately 20 ± 2 μm. All the fibers except the PLA/PBS 10% fiber exhibit a uniform phase in their cross-sectional surface. In contrast, the PLA/PBS 10% fiber displays clear phase separation on the cross-sectional surface, indicating the low compatibility between PBS and PLA due to the higher content of PBS.

#### 3.2.2. Effects of PBS Content on the Crystallinity of PLA/PBS Blend Fibers

In this study, the PLA/PBS blend fibers were spun under optimized conditions, including extrusion temperature, extrusion speed, spinning temperature, spinning speed, and draw ratio. These conditions were meticulously selected based on the rheological and thermal properties of the PLA/PBS blends. It was expected that the PLA/PBS blend fibers would exhibit changes in crystallinity compared to the PLA/PBS blends. To understand the factors contributing to these phenomena, we carried out a DSC analysis and 2D-WAXD analysis.

**Figure 8:**
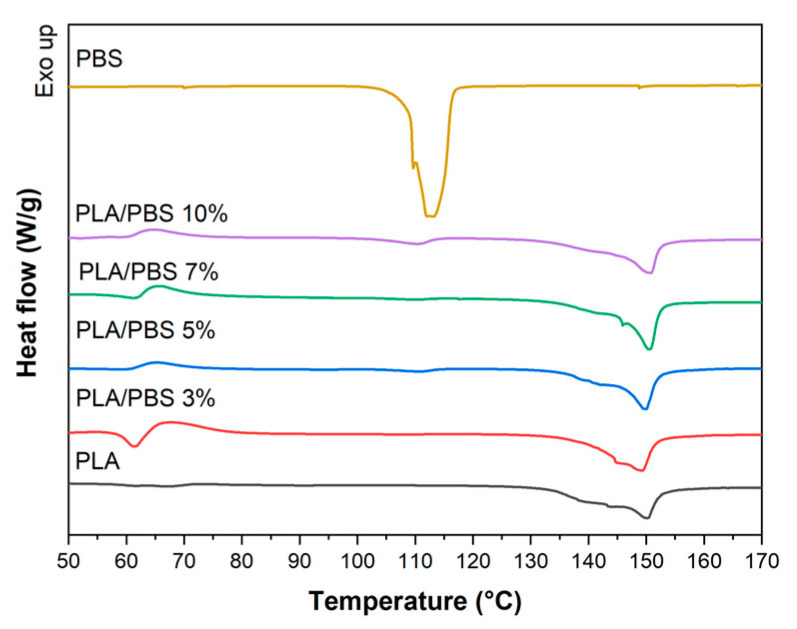
DSC curves of the PLA/PBS blend fibers.

Figure 8 displays a slightly elevated endothermic peak above the glass transition temperature for the PLA/PBS blend fibers. This peak corresponds to the relaxation of ordered polymer chains during the stretching of the drawing process. The intensity of this peak varies based on the PBS content within the PLA/PBS blend fibers.

The calculated degree of crystallinity in the PLA/PBS blends indicates a notable enhancement due to the addition of PBS compared to pure PLA. As listed in Table 5, there is an improvement of over 2.8 times in the degree of crystallinity across all PLA/PBS blend fibers compared to the PLA/PBS blends. Specifically, in the case of PLA/PBS 7%, the degree of crystallinity increases from 7.69% to 37.87%. It is worth noting that all PLA/PBS blend fibers exhibited a higher degree of crystallinity compared to pure PLA fiber. This suggests that adding PBS enhances chain mobility and improves chain packing under the same drawing conditions.

**Figure 9:**
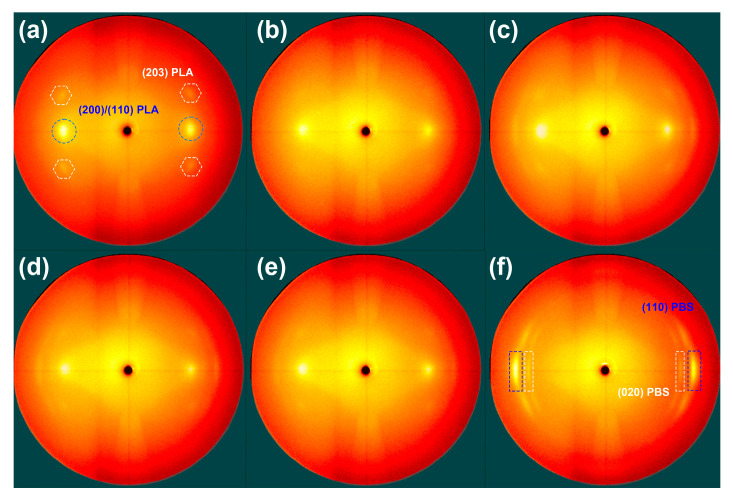
The 2D-WAXD images for the PLA, PBS, and PLA/PBS blend fibers. (**a**) PLA fiber, (**b**) PLA/PBS 3% fiber, (**c**) PLA/PBS 5% fiber, (**d**) PLA/PBS 7% fiber, (**e**) PLA/PBS 10% fiber, and (**f**) PBS fiber. The blue dot circles and squares represent the (200)/(110) plane of PLA. The white dot hexagons and squares represent the (203) plane of PLA.

Figure 9 illustrates the 2D-WAXD images capturing the distinctive diffraction patterns of the PLA, PBS, and PLA/PBS blend fibers. In the case of the PLA fibers, the diffraction pattern observed at *q* = 11.8 nm^−1^ is designated as the (200)/(110) plane of PLA, represented by two solid points along the equator. Additionally, the pattern observed at *q* = 13.5 nm^−1^ is assigned as the (203) plain of PLA, forming a distinctive set of four solid points. These patterns strongly suggest a directional alignment of the crystal axis, specifically along the (200)/(110) and (203) crystallographic planes within the α/α’ crystal form of PLA [10]. In contrast, the PBS fibers exhibit arc-like diffraction patterns. Notably, the (110) diffraction emerges at *q* = 15.56 nm^−1^, the (021) diffraction is observed at *q* = 15.28 nm^−1^, and the (020) diffraction appears at *q* = 13.81 nm^−1^ [17]. With increasing PBS content within the PLA/PBS blend fibers, the peak characteristics of PBS become more pronounced, leading to heightened intensity within the 2D-WAXD images. Additionally, there is evidence to suggest that PBS aids in establishing a well-oriented lattice in the PLA fibers.

**Figure 10:**
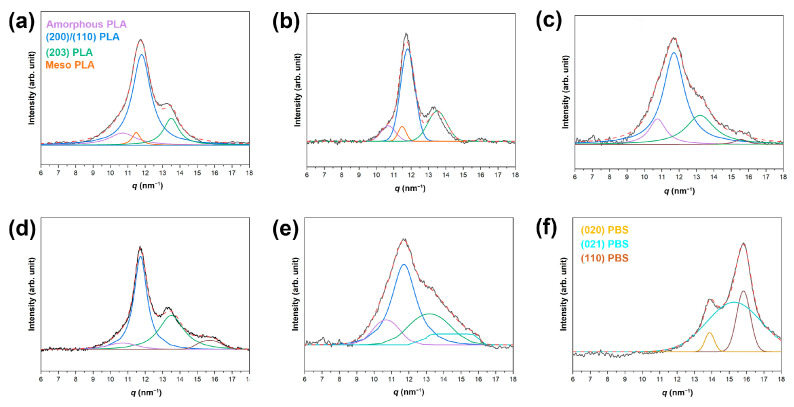
Deconvolution curves of 1D-WAXD profiles for (**a**) the PLA fiber, (**b**) the PLA/PBS 3% fiber, (**c**) the PLA/PBS 5% fiber, (**d**) the PLA/PBS 7% fiber, (**e**) the PLA/PBS 10% fiber, and (**f**) the PBS fiber. The Gaussian + Lorentzian function is used for the deconvolution of the peaks. The red dotted line represents the sum of the deconvolution peaks. The black line represents the original peak.

To determine the crystallinity in the PLA/PBS blend fibers, we calculated the ratio between the summation of areas corresponding to crystalline regions within the PLA/PBS blend fibers. The 1D-WAXD patterns reveal crystalline peaks corresponding to the (200)/(110) and (203) planes in both the PLA fibers and the PLA/PBS 3% fibers (Figure 10). Alongside these, there is evidence of the amorphous phase of PLA and mesophase peaks at *q* = 10.68 nm^−1^ and 11.48 nm^−1^, respectively. As shown in Table 6, the calculated crystallinity indicates an increase in the degree of crystallinity of PLA/PBS fibers as the PBS content rises to 7 wt.%. This observation aligns consistently with the findings from DSC results.

**Figure 11:**
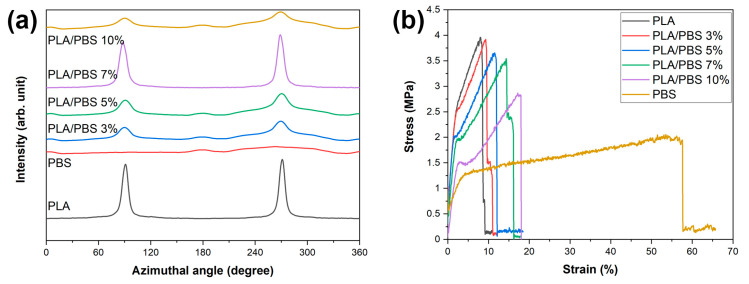
(**a**) Azimuthal profiles across the (200)/(110) plane of PLA. (**b**) Stress–strain curves of the PLA/PBS blend fibers.

Table 7 presents the calculated degree of fiber orientation, confirming the difference in orientation between the PLA and PLA/PBS blend fibers. The degree of orientation was calculated using two functions, which are the crystalline orientation function and Herman’s orientation function, respectively. An essential factor in the crystalline orientation function is the full width at half maximum (*W_h_*) in the azimuthal direction of the selected diffraction peak. The related equations are listed as follows (Figure 11a).

(5) $$f_{c}=\frac{180^{{}^{\circ}}-W_{h}}{180}$$

The *f_c_* value of PLA is 0.951, which means that the PLA fiber is highly aligned along with the (200)/(110) plane direction of PLA. However, all PLA/PBS blend fibers exhibit lower *f*_c_ values than the PLA fiber. This indicates a more downward orientation of the α/α’ crystal form of PLA. A similar trend was observed by the calculated result of Herman’s orientation parameter.

(6) $$f_{H}=\frac{3({cos}^{2}\varphi {)}_{hkl}-1}{2}$$

where ${cos}^{2}\varphi$ is an orientation factor defined as follows.

(7) $${(cos}^{2}\varphi )=\frac{\int_{0}^{\pi /2}I\left(\varphi \right)cos^{2}\varphi sin\varphi d\varphi }{\int_{0}^{\pi /2}I\left(\varphi \right)sin\varphi d\varphi }$$

where *I(φ)* represents the scattering intensity along the angle φ. When the blending materials in the fibers align parallel to the reference axis, they exhibit an *f*_*H*_ value of 1. In contrast, perpendicular alignment to the reference axis results in *f*_*H*_ values of −0.5, while random orientation corresponds to an *f*_*H*_ value of 0.

The degree of orientation of the PLA fibers displays a similar value of 0.719 with PLA/PBS 7%. However, other PLA/PBS blend fiber samples show a reduction in Herman’s orientation parameter in the order of PLA/PBS 7% ≈ PLA > PLA/PBS 5% > PLA/PBS 3% > PLA/PBS 10% > PBS. This is due to the specific content of PBS that improves the orientation of the PLA/PBS blend fibers.

#### 3.2.3. Mechanical Properties of PLA/PBS Blend Fibers

The mechanical characteristics of the PLA/PBS blend fibers were evaluated through tensile strength tests. Figure 11b demonstrates the correlation between tensile strength and elongation at break based on the PBS content in the PLA/PBS blend fibers. A summary of the mechanical test outcomes is provided in Table 7. Specifically, the PLA fiber exhibited a tensile strength of 3.58 MPa and an associated elongation at break of 10.24%. Conversely, the PBS fiber displayed a comparatively lower tensile strength of 1.66 MPa but demonstrated a higher elongation at break of 58.61%. As the PBS content increased in the PLA/PBS blend fibers, there was a gradual decrease in tensile strength to 2.55 MPa, accompanied by a rise in elongation at break, reaching up to 19.22% for the PLA/PBS 10% fibers. This suggests that adding PBS to PLA fibers enhances the elongation at break of the PLA/PBS blend fibers due to the flexible molecular chain of PBS. Notably, among the samples, the PLA/PBS 7% blend fiber showed an improvement in elongation at break (approximately 16.58%) with a slight decrease in tensile strength (approximately 3.16 MPa) [27,28,29].

## 4. Conclusions

In conclusion, our investigation into incorporating PBS content into PLA to develop blend fibers revealed notable outcomes. The rheological properties, crystallinity, and phase separation dynamics within the PLA/PBS blends were influenced by varying PBS contents. Specifically, there is an optimal PBS content for fabricating the PLA/PBS blends, resulting in fibers with improved crystallinity and mechanical properties. Interestingly, the PLA/PBS blend fiber with a weight ratio of 93:7 between PLA and PBS exhibited higher crystallinity and Herman’s orientation factor. This is attributed to PBS acting as a nucleating agent and achieving proper orientation during the drawing process. The mechanical strength of the PLA/PBS 7% fiber exhibited a value similar to the pure PLA fiber (~3.58 MPa) while also improving elongation at break (~16.58%). This observation underscores the potential for enhancing the mechanical properties of PLA fibers by strategically incorporating PBS. Our study contributes valuable insight into the fabrication and properties of PLA/PBS blend fibers, opening avenues for further exploration and optimization in textile applications.

## Figures and Tables

**Table 1:** Material properties of PLA and PBS.

Property	PLA	PBS
Glass transition temperature (°C)	55–60	−32
Melting point (°C)	150–175	114
Modulus of elasticity (MPa)	3500–4150	550–700
Tensile strength (MPa)	50–70	34
Elongation at break (%)	4–7	560
Biodegradability at 70 °C	Yes	Yes
Biodegradability at 30 °C	No	Yes

**Table 2:** Melt flow index of PLA, PBS, and PLA/PBS blends.

Sample	Melt Flow Index (g/10 min)		
	At 190 °C	At 210 °C	At 230 °C
PLA	3.36	8.47	10.69
PBS	22.62	34.51	48.26
PLA/PBS 3%	3.42	8.65	11.63
PLA/PBS 5%	3.42	8.68	12.13
PLA/PBS 7%	3.44	8.91	12.25
PLA/PBS 10%	3.47	9.36	13.61

**Table 3:** Power law fitting parameters for the melt shear viscosity at 230 °C.

Sample	*K* (Pa·s)	*n*
PLA	159.2	0.97
PBS	0.09	0.11
PLA/PBS 3%	30.0	0.86
PLA/PBS 5%	7.2	0.84
PLA/PBS 7%	4.9	0.82
PLA/PBS 10%	0.2	0.29

**Table 4:** Thermal properties of PLA in the PLA/PBS blends.

Sample	T_gPLA_ (°C)	T_ccPLA_ (°C)	T_m1PLA_ (°C)	T_m2PLA_ (°C)	ΔH_ccPLA_ (J g^−1^)	ΔH_mPLA_ (J g^−1^)	χ_cPLA_ (%)
PLA	53.36	100.68	143.91	152.54	30.74	34.31	3.84
PBS	-	-	-	-	-	-	-
PLA/PBS 3%	54.30	101.69	144.38	152.56	19.05	30.91	13.15
PLA/PBS 5%	54.43	105.70	145.21	152.66	17.34	29.09	13.27
PLA/PBS 7%	53.96	102.23	144.19	151.36	19.55	26.20	7.69
PLA/PBS 10%	53.66	101.97	143.75	150.36	17.33	23.38	7.23

**Table 5:** Thermal properties of the PLA/PBS blend fibers.

Sample	T_gPLA_ (°C)	T_mPLA_ (°C)	ΔH_mPLA_ (J g^−1^)	χ_cPLA_ (%)	T_mPBS_ (°C)	ΔH_mPBS_ (J g^−1^)	χ_cPBS_ (%)
PLA	59.48	150.17	32.59	35.04	-	-	-
PBS	-	-	-	-	113.08	72.51	34.53
PLA/PBS 3%	59.27	149.49	33.13	36.73	108.57	0.51	8.10
PLA/PBS 5%	59.12	149.89	33.01	37.36	110.79	1.52	14.48
PLA/PBS 7%	58.62	150.59	32.75	37.87	109.80	2.15	14.63
PLA/PBS 10%	53.66	150.77	29.53	35.28	110.21	3.61	17.19

**Table 6:** The crystallinity of the PLA/PBS blend fibers.

Sample	X_c(200)/(110)PLA_ (%)	X_c(203)PLA_ (%)	X_amorPLA_ (%)	X_mesoPLA_ (%)	X_(110)PBS_ (%)	X_(020)PBS_ (%)	X_(021)PBS_ (%)	X_c_^PLA^ (%)
PLA	41.19	24.05	23.51	11.25	-	-	-	65.24
PBS	-	-	-	-	4.84	22.71	72.45	-
PLA/PBS 3%	36.04	32.15	23.60	8.21	-	-	-	68.19
PLA/PBS 5%	37.08	33.61	24.62	-	3.89	-	-	70.69
PLA/PBS 7%	37.62	38.51	14.23	-	9.64	-	-	76.13
PLA/PBS 10%	27.76	34.12	23.00	-	-	-	15.12	61.88

**Table 7:** Degree of orientation with a specific direction of (200)/(110) plane of PLA and mechanical properties of the PLA/PBS blend fibers.

Sample	*W_h_*	*f_c_*	*f_H_*	Tensile Strength (MPa)	Elongation at Break (%)
PLA	8.865	0.951	0.718	3.58 ± 0.51	10.24 ± 0.40
PBS	164.24	0.085	0.016	1.66 ± 0.16	58.61 ± 0.21
PLA/PBS 3%	32.41	0.820	0.321	3.55 ± 0.35	11.63 ± 0.18
PLA/PBS 5%	29.06	0.839	0.541	3.28 ± 0.21	12.36 ± 0.28
PLA/PBS 7%	10.58	0.941	0.719	3.16 ± 0.14	16.58 ± 0.36
PLA/PBS 10%	38.90	0.784	0.265	2.55 ± 0.27	19.22 ± 0.49

## Data Availability

The data presented in this study are available on request from the corresponding author.
